# 苹果酸舒尼替尼治疗多次复发的晚期非小细胞肺癌疗效与安全性分析

**DOI:** 10.3779/j.issn.1009-3419.2013.10.04

**Published:** 2013-10-20

**Authors:** 镨元 邢, 峻岭 李, 远凯 石

**Affiliations:** 100021 北京，北京协和医学院，中国医学科学院肿瘤医院内科，抗肿瘤分子靶向药物临床研究北京市重点实验室 Department of Medical Oncology, Cancer Institute/Hospital, Peking Union Medical College & Chinese Academy of Medical Sciences; Beijing Key Laboratory of Clinical Study on Anticancer Molecular Targeted Drugs, Beijing 100021, China

**Keywords:** 肺肿瘤, 苹果酸舒尼替尼, 复发, 酪氨酸激酶抑制剂, Lung neoplsms, Sunitinib, Recurrence, Tyrosine kinase inhibitors

## Abstract

**背景与目的:**

晚期非小细胞肺癌是一种无法治愈的常见恶性肿瘤。对于多程治疗后复发的患者目前尚无标准的治疗方法。本文分析了苹果酸舒尼替尼单药治疗多次复发的晚期非小细胞肺癌的疗效及安全性。

**方法:**

回顾性分析我科2011年1月-2012年12月采用苹果酸舒尼替尼37.5 mg/d持续给药的方法治疗的17例多程治疗后复发的晚期非小细胞肺癌患者的近期疗效、毒副反应及无进展生存时间。

**结果:**

17例患者中部分缓解1例（5.9%），稳定7例（41.2%），疾病进展9例（52.9%），客观缓解率5.9%，疾病控制率47.1%，中位无进展生存为4.4个月（95%CI: 4.05-7.46）。全组患者治疗耐受良好，3级/4级不良反应仅表现为手足皮肤反应（5.9%），其余药物相关不良事件均为1/2级。

**结论:**

苹果酸舒尼替尼37.5 mg/d持续治疗多程治疗后复发的晚期非小细胞肺癌可取得较好的客观疗效，耐受良好。

## Abstract (en)

**Background and objective:**

Advanced non-small cell lung cancer (NSCLC) is a common malignancy that is incurable. No standard treatment exists for recurrent patients. This article analyzed the efficacy and safety of sunitinib (37.5 mg qd) on a continuous daily dosing (CDD) schedule in treating recurrent advanced NSCLC.

**Methods:**

We retrospectively analyzed the short-term efficacy and toxicity of sunitinib CDD in treating 17 patients who had previously undergone multiple cycles of therapy for advanced NSCLC in our hospital from January 2011 to December 2012. Treatment-related survival was also analyzed.

**Results:**

Among the 17 patients, the best overall response was partial response in 1 patient (5.9%), stable disease in 7 patients (41.2%), and progressive disease in 9 patients (52.9%). The overall response rate was 5.9%, and the disease control rate was 47.1%. The median progression-free survival was 4.4 months (95%CI: 4.05-7.46). The main grade 3/4 toxicity was hand-foot skin reaction.

**Conclusion:**

Sunitinib (37.5 mg QD) CDD enabled good objective response in advanced NSCLC patients who had previously received multiple cycles of treatment and was well tolerated.

非小细胞肺癌（non-small cell lung cancer, NSCLC）是肺癌中最常见的病理类型，其发病率约占肺癌总发病的75%-80%。初诊时约60%已为晚期，无法彻底治愈，因此需要经历较长期的临床治疗以达到延长生存时间的目的。虽然近十年随着分子靶向药物的发展，晚期NSCLC患者的生存得到明显改善，但是对于部分无明确治疗靶点或多程治疗后复发的患者，仍然需要继续积极寻找新的、有效的治疗手段。

苹果酸舒尼替尼（sunitinib, sutent）一种新型吲哚酮类口服、选择性多靶点酪氨酸激酶抑制剂，除抑制血管内皮细胞生长因子受体（vascular endothelial growth factor receptor, VEGFR）-1, 2, 3、血小板源性生长因子受体（platelet derived growth factor receptor, PDGFR）-α, β的活性外，同时也抑制几种其他相关的酪氨酸激酶的活性^[1-5]^，具有抗血管生成和抗肿瘤活性的双重作用。临床前期研究^[2, 3]^提示，舒尼替尼能够有效的抑制人类NSCLC异种移植模型的生长。多项临床研究评估了舒尼替尼在晚期NSCLC治疗中的作用，初步显示其在多线治疗后的晚期NSCLC中仍能够取得一定疗效、改善患者生存，且毒性可耐受，为标准治疗失败后的晚期NSCLC提供了一种新的治疗选择。本文回顾性分析了2011年1月-2012年12月间中国医学科学院北京协和医学院肿瘤医院内科收治的17例接受单药舒尼替尼治疗的复发/难治性晚期NSCLC患者临床病理资料，希望能够为后续临床治疗提供参考。

## 1 材料与方法

### 1.1 临床资料

2011年1月-2012年12月我科室收治17例经过多线治疗进展的晚期NSCLC患者，给予单药舒尼替尼（37.5 mg/d）治疗。全部患者临床病理资料完整，按计划定期随访至影像学评估为进展。17例患者中男性8例，女性9例；年龄范围37岁-72岁，中位年龄53岁；ECOG 0分-1分；吸烟7例，不吸烟10例；全组患者治疗前临床分期均为Ⅳ期；病理类型13例为腺癌，1例为腺鳞癌，1例为NSCLC未分型，1例为大细胞癌，1例为肉瘤样癌；患者既往接受过3线-8线方案的全身治疗，中位既往接受过4线方案治疗；15例患者接受过表皮生长因子受体酪氨酸激酶抑制剂（epidermal growth factor receptor tyrosine kinase inhibitors, EGFR-TKIs）治疗；11例*EGFR*基因状况不明确，6例接受过*EGFR*基因检测，其中敏感突变型2例，野生型4例；主要转移部位包括肺（11/17, 64.7%）、脑（7/17, 41.2%）、骨（6/17, 35.3%）、肝脏（3/17, 17.6%）、肾上腺（4/17, 23.5%）、其他（12/17, 70.6%）。既往化疗中含有吉西他滨13例（76.5%）、培美曲赛14例（82.4%）、多西他赛10例（58.8%）、紫杉醇7例（41.2%）、顺铂16例（94.1%）、卡铂11例（64.7%）、其它化疗药物9例（52.9%）（表 1）。

**1:** 患者基线特征 Baseline demographic and disease characteristics

Characteristics	Sunitinib (*n*=17)
Age in years, median (range)	53 (7-72)
Male/female, *n* (%)	8 (47.1)/9 (52.9)
ECOG PS 0/1, *n* (%)	11 (64.7)/6 (35.3)
Smokiing/non-smoking, *n* (%)	7 (41.2)/10 (58.8)
NSCLC histology, *n* (%) Adenocarcinoma Squamous cell carcinoma Large cell carcinoma Other	13 (76.5) 1 (5.9) 1 (5.9) 2 (11.7)
Metastatic sites, *n* (%) Lung/CNS/bone/liver/adrenal/other	11 (64.7)/7 (41.2)/6 (35.3)/3 (17.6)/4 (23.5)/12 (70.6)
Previous chemotherapy, *n* (%) Gemcitabine Pemetrexed Docetaxel Paclitaxel Cisplatin Carboplatin Other	13 (76.5) 14 (82.4) 10 (58.8) 7 (41.2) 16 (94.1) 11 (64.7) 9 (52.9)
Maximum number of previous regimens, *n* (%) Chemotherapy 2/3/ > 3 EGFR-TKIs 1/2	5 (29.4)/5 (29.4)/7 (41.2) 10 (58.8)/5 (29.4)
ECOG PS: Eastern Cooperative Oncology Group performance status; NSCLC: non-small cell lung cancer; CNS: central nervous system; EGFR-TKIs: epidermal growth factor receptor tyrosine kinase inhibitors.	

### 1.2 方法

#### 1.2.1 治疗方案设计

治疗药物为苹果酸舒尼替尼，初始剂量37.5 mg/d，口服，4周为1治疗周期。药物剂量根据不良反应进行调整，首次减量为25 mg/d，第2次减量剂量可调整为12.5 mg/d。在治疗阶段，患者能够每4周接受1次治疗访视，用药4周后进行首次基于疗效评价标准（Response Evaluation Criteria in Solid Tumors, RECIST）的影像学（CT/MRI）评估，之后每8周接受1次基于RECIST标准的影像学（CT/MRI）评估。患者均定期进行肿瘤评估直至出现疾病进展，随后进行生存随访直至死亡、失访。

#### 1.2.2 疗效及不良反应评价

疗效的客观判断标准按照实体瘤的评价标准（RECIST 1.1）进行评定，观察指标包括完全缓解（complete response, CR）、部分缓解（partial response, PR）、稳定（stable disease, SD）和进展（progressive disease, PD），客观有效率（objective response rate, ORR）和疾病控制率（disease control rate, DCR）。无疾病进展生存时间（progression-free survival, PFS）定义为从首次给药至有客观证据证实的疾病进展的时间。不良反应评价按NCI-CTC（National Cancer Institute-Common Toxicity Criteria）3.0版本标准进行评价。

### 1.3 统计学方法

本组数据使用SPSS 16.0统计软件，采用*Kaplan-Meier*法进行生存分析，*P* < 0.05为差异有统计学意义。

## 2 结果

### 2.1 近期疗效及生存结果

2011年1月-2012年12月17例患者接受治疗时间为4周-36周，平均12.9周，中位治疗10周，2例（11.8%）患者因药物相关不良反应减量，其中1例减量至25 mg/d，1例多次减量至12.5 mg/d。全组患者均可进行肿瘤疗效评估。PR 1例（5.9%），SD 7例（41.2%），PD 9例（52.9%），ORR（CR+PR）5.9%，DCR（CR+PR+SD）47.1%。随访时间截至2013年4月30日，中位随访时间8个月（3个月-26个月），中位PFS为4.4个月（图 1）。总生存期随访全组5例（29.4%）患者因肿瘤进展死亡，12例（70.6%）患者生存。

**1:**
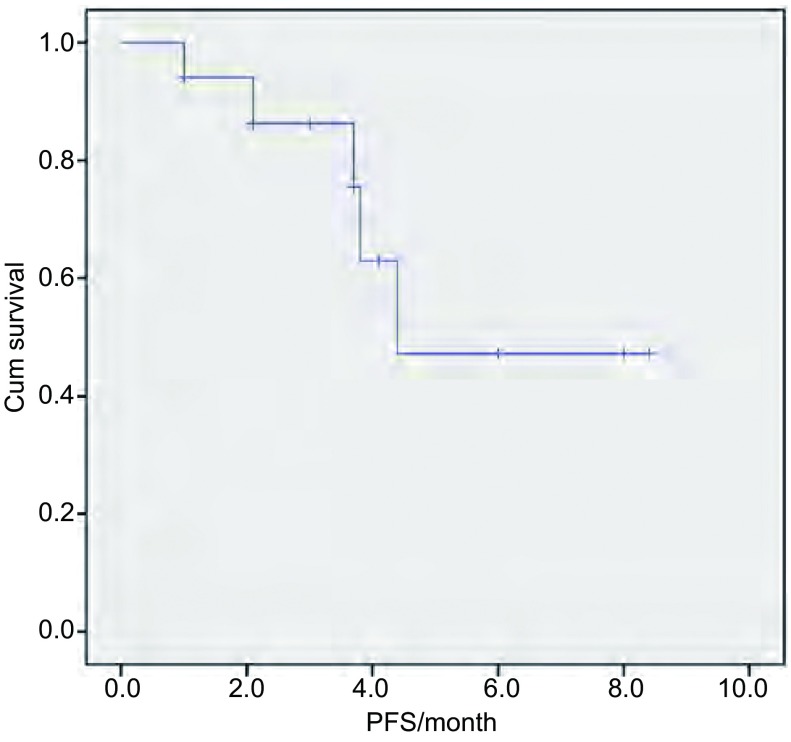
患者无进展生存时间的生存曲线 *Kaplan-Meier* survival curves of progression-free survival (PFS) of patients

### 2.2 不良反应

本组患者使用单药舒尼替尼治疗主要不良反应表现为中性粒细胞计数减低、血小板计数减低、乏力、手足皮肤反应等，3级/4级不良反应少见，多为1级/2级不良反应（表 2）。

**2:** 常见不良反应发生率 Incidence of the common treatment-related adverse events

Adverse event	Total grades, *n* (%)	Grade 3/4, *n* (%)
Neutropenia	5 (29.4)	0 (0)
Thrombocytopenia	3 (17.6)	0 (0)
Fatigue/asthenia	8 (47.1)	0 (0)
Hand-foot skin reaction	7 (41.2)	1 (5.9)
Nausea	4 (23.5)	0 (0)
Anorexia	4 (23.5)	0 (0)
Diarrhea	1 (5.9)	0 (0)
ALT increased	1 (5.9)	0 (0)
Hypertension	2 (11.8)	0 (0)
Hypokalemia	2 (11.8)	0 (0)

## 3 讨论

苹果酸舒尼替尼是新型多靶点酪氨酸激酶抑制剂，理论上讲它能够从多环节抑制肿瘤细胞增殖及肿瘤微环境形成，弥补了使用单靶点药物后其他通路上的肿瘤逃逸。但是由于多方面因素限制，目前尚无多靶点药物能够被批准用于NSCLC，仅限于临床研究^[6-15]^。目前进行的多项舒尼替尼单药治疗晚期NSCLC临床研究结果显示患者获得的ORR为1.6%-11.1%，PFS为9.4周-12.1周，OS为23.4周-26周。种族、性别、吸烟状况、*EGFR*/*KRAS*基因状态等因素均与疗效及预后无明显相关性。一项舒尼替尼联合厄洛替尼对比单药厄洛替尼二/三线治疗NSCLC的Ⅱ期临床试验（SUN1058）进行了生物标志物分析：在组织学标本指标中，PDGFRα mRNA低表达患者的疗效优于PDGFRα mRNA高表达的患者（HR=0.386, *P* < 0.05）；在血清蛋白分子标志物检测中，发现类似sVEGFR-3、sVEGFR-2以及VEGFC水平的变化与舒尼替尼疗效的相关性，譬如90%疾病获得控制的患者基础血浆sVEGFR-3水平 < 51, 200 pg/mL。其他实体瘤的临床研究^[17]^中也验证了sVEGFR-3有可能成为抗血管生成的酪氨酸激酶抑制剂疗效判定的潜在生物标志物。

舒尼替尼给药方式主要有两种：①50 mg/d，连续服药4周，休息2周；②37.5 mg/d，连续服药。从所得结果看出，两种给药方式mPFS相似，前者获得了较好的ORR，后者在中位总生存（median overall survival, mOS）上更具优势且耐受性更好。本组病例均为多线治疗后、缺乏标准治疗方案、体力状况良好的晚期NSCLC患者，采用舒尼替尼37.5 mg，qd，不间断服药的探索性治疗。从近期疗效上看，虽然本组病例为多线治疗后，但是ORR为5.88%，DCR为47.1%，与既往研究相仿，可能得益于患者治疗前仍然保持着良好的体力状态。毒性反应中最严重影响患者生活质量的是手足皮肤反应（hand-foot skin reaction, HFSR），并且成为影响患者医从性的主要原因。本组研究中全部等级的HFSR发生率为41.2%（42.9%为Ⅱ级），3级/4级HFSR发生率为5.9%（1/17），这例患者因合并3级HFSR而2次调整药物剂量。美国学者针对多靶点药物引起的HFSR进行了相关研究，结果提示HFSR能够明显影响患者生活质量（health-related quality of life, HRQoL）^[12]^。乏力是本组病例中发生率最高的药物相关不良反应，但75%为1级，未对患者生活质量及治疗造成明显影响。可能由于既往多线治疗影响，血液学毒性发生概率与既往随机研究相比较略高，但均可耐受。因为舒尼替尼不良反应进行药物减量的发生频率为17.6%（3/17）。

除舒尼替尼单药治疗外，近期报告的几项临床研究^[11, 13-17]^结果提示与单靶点药物或化疗联合在二/三线治疗晚期NSCLC能够取得更好的疗效，但是毒性反应似乎较单药明显，因此对于体力状况允许的患者我们亦可以考虑联合治疗。其中入组病例数最多的一项双盲随机Ⅲ期研究SUN1087最新结果显示，舒尼替尼联合厄洛替尼在PFS（3.6个月*vs* 2.0个月，*P*=0.002, 3）及ORR（10.6% *vs* 6.9%, *P*=0.047, 1）上均优于单药厄洛替尼，两组OS未看到统计学差异（9.0个月*vs* 8.5个月，*P*=0.138, 8）。由于这项研究开始较早，未根据*EGFR*基因突变状况将患者进一步分层，因此无法确定EGFR-TKIs优势人群是否同样能够从联合治疗中获益，抑或是EGFR野生型患者获益更多。

总之，以苹果酸舒尼替尼为代表的多靶点抗肿瘤药物治疗NSCLC，在疗效与耐受性、给药剂量与时间的选择、与传统化疗或单靶点药物的联合、药物获益优势人群等问题上仍然需要进一步大样本Ⅲ期随机临床研究验证。
